# Targeting METTL3 as a checkpoint to enhance T cells for tumour immunotherapy

**DOI:** 10.1002/ctm2.70089

**Published:** 2024-11-20

**Authors:** Kaixin Wu, Sa Li, Guangliang Hong, Hongzhi Dong, Tongke Tang, He Liu, Lingmei Jin, Siyuan Lin, Jingyun Ji, Mingli Hu, Shuntian Chen, Haoyuan Wu, Guanzheng Luo, Haoyuan Wu, Xiangqian Kong, Jiekai Chen, Jiangping He, Hongling Wu

**Affiliations:** ^1^ Center for Cell Lineage and Atlas Bioland Laboratory, Guangzhou Regenerative Medicine and Health GuangDong Laboratory Guangzhou China; ^2^ The Fifth Affiliated Hospital of Guangzhou Medical University Guangzhou China; ^3^ Joint School of Life Sciences Guangzhou Institutes of Biomedicine and Health Chinese Academy of Sciences Guangzhou Medical University Guangzhou China; ^4^ Center for Cell Lineage and Development Guangdong Provincial Key Laboratory of Stem Cell and Regenerative Medicine Guangzhou Institutes of Biomedicine and Health Chinese Academy of Sciences Guangzhou China; ^5^ University of Chinese Academy of Sciences Beijing China; ^6^ Guangzhou National Laboratory Guangzhou China; ^7^ MOE Key Laboratory of Gene Function and Regulation Guangdong Province Key Laboratory of Pharmaceutical Functional Genes State Key Laboratory of Biocontrol School of Life Sciences Sun Yat‐Sen University Guangzhou China; ^8^ Science and Technology Innovation Center Guangzhou University of Chinese Medicine Guangzhou China; ^9^ Centre for Regenerative Medicine and Health Hong Kong Institute of Science & Innovation Chinese Academy of Sciences Hong Kong SAR China; ^10^ State Key Laboratory of Respiratory Disease National Clinical Research Center for Respiratory Disease National Center for Respiratory Medicine Guangzhou Institute of Respiratory Health The First Affiliated Hospital of Guangzhou Medical University Guangzhou China

**Keywords:** B16 melanoma, immunotherapy, METTL3, N6‐methyladenosine (m^6^A), T‐cell function, YTHDF2

## Abstract

**Background:**

Immunotherapy has emerged as a crucial treatment modality for solid tumours, yet tumours often evade immune surveillance. There is an imperative to uncover novel immune regulators that can boost tumour immunogenicity and increase the efficacy of immune checkpoint blockade (ICB) therapy. Epigenetic regulators play critical roles in tumour microenvironment remodelling, and N6‐methyladenosine (m^6^A) is known to be involved in tumourigenesis. However, the role of m^6^A in regulating T‐cell function and enhancing anti‐tumour immunity remains unexplored.

**Methods:**

Several cancer cell lines were treated with STM2457, an enzymatic inhibitor of RNA m^6^A methyltransferase METTL3, and explored the transcriptome changes with RNA sequencing (RNA‐seq). We then utilised mouse melanoma (B16) and mouse colorectal adenocarcinoma (MC38) models to investigate the effects of METTL3 inhibition on immunotherapy, and analysed the dynamics of the tumour microenvironment via single‐cell RNA‐seq (scRNA‐seq). Furthermore, in vitro and in vivo T‐cell cytotoxicity killing assay and CRISPR Cas9‐mediated m^6^A reader YTHDF1‐3 knockout in B16 were performed to assess the role and the molecular mechanism of RNA m^6^A in tumour killing. Finally, the efficacy of METTL3 inhibition was also tested on human melanoma model (A375) and human T cells.

**Results:**

We demonstrate that inhibiting METTL3 augments tumour immunogenicity and sustains T‐cell function, thereby enhancing responsiveness to ICB therapy. Mechanistically, METTL3 inhibition triggers an interferon response within tumour cells, amplifying the anti‐tumour immune response, along with deletion of the m^6^A reader protein YTHDF2 in tumours inhibiting major histocompatibility complex (MHC)‐I degradation. Remarkably, these anti‐tumour effects are reliant on the immune system. Specifically, METTL3 inhibition enhances interferon‐gamma (IFNγ) and granzyme B (GzmB) expression, thereby strengthening T‐cell killing ability, and concurrently dampening the expression of exhaustion‐related genes.

**Conclusion:**

Targeting METTL3 enhances anti‐tumour immunity by boosting T‐cell cytotoxicity and reversing T‐cell exhaustion. Our study positions METTL3 as an epigenetic checkpoint, highlighting the potential of targeting METTL3 to invigorate intrinsic anti‐tumour defenses and overcome immune resistance.

**Key points:**

- Targeting METTL3 augments tumour cell immunogenicity and sustains T‐cell function.
- T cell with METTL3 inhibition can reverse T‐cell exhaustion, and promote expression of IFNγ and GzmB, thereby enhancing cytotoxicity in anti‐PD‐1 therapy.
- YTHDF2 deletion in tumours prolong the lifespan of MHC‐I mRNAs.

## Graphical abstract

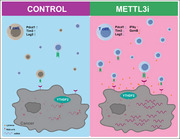

## 1 BACKGROUND

N6‐methyladenosine (m^6^A) is the most abundant internal modification in mRNA.^1^ And it is a dynamic and reversible chemical modification methylated by a METTL3/METTL14 complex, recognised by readers such as YTHDF1/2/3, and erased by proteins such as FTO and ALKBH5.^2^ RNA m^6^A modification is involved in a series of mRNA metabolism processes, including splicing, translation, stability, degradation and nucleation.^3^ These RNA metabolic processes regulated and influenced by m^6^A play important roles in cell fate determination and in both physiological and pathological conditions, especially in the initiation and progression of cancer.^4^, ^5^

Therapies on immune checkpoint blockade (ICB) has revolutionised cancer treatment, but many patients do not respond or resistance to ICB. Epigenetic regulatory abnormalities, as one of the factors contributing to tumour development, have shown promising application prospects when combined with immune checkpoint inhibitors and epigenetic inhibitors. The use of epigenetic inhibitor such as DNMT1, EZH27, SETDB1, etc., have been reported to improve anti‐tumour immunity, by increasing the interferon response within tumour cells, upregulating the expression of antigen presenting genes, thereby transforming ‘immune cold tumours’ into ‘immune hot tumours’.^6^, ^7^ RNA m^6^A modification can directly regulate immune pathway‐related genes in tumour cells, and the interplay between m^6^A and immunotherapy is of great significance. It is reported that RNA m^6^A modification regulators can involve in regulating PD‐1/PD‐L1 expression in various tumours.^8^ Meanwhile, RNA m^6^A intervention combined with PD‐1 has shown positive effects in immunotherapy.^9^, ^10^ Several research have reported that aberrant expression of m^6^A regulators, including ‘writers’, ‘readers’ and ‘erasers’, contribute to carcinogenesis, progression and drug resistance in various cancers.^8^ ALKBH5/FTO deletion, for instance, can enhance the efficacy of anti‐PD‐1 therapy.^11^, ^12^ YTHDF1 impairs anti‐tumour immunity via an m^6^A‐p65‐CXCL1/CXCR2 axis and serves as a therapeutic target for ICB in colorectal cancer.^13^ In addition, the knockout (KO) of m^6^A methyltransferase METTL3 or the use of inhibitor can also enrich immune pathway in tumour cells and upregulate the expression of antigen presentation, thereby enhancing the effect of ICB treatment.^14^, ^15^ In general, current research on the mechanism of RNA m^6^A in tumour immunity predominantly emphasises the impact of RNA m^6^A regulation on the expression of immune‐related gene or antigen presentation within tumour cells. However, investigations of RNA m^6^A on the tumour microenvironment and among immune cells themselves are comparatively limited. The mechanism by which m^6^A modification on tumours and the immune environment, respectively, for immunotherapy have yet to be comprehensively delineated.

In mammals, specific KO of METTL3 in mouse embryonic stem cells can cause haematopoietic failure and perinatal death, indicating that RNA m^6^A modification is crucial for the normal development of the haematopoietic system.^16^ Among them, T cells play an important role in maintaining the homeostasis of the immune system. Research shown that KO of METTL3 in CD4^+^ T cells leads to a disruption in the lymphocyte balance of the immune system. The absence of METTL3 impairs the proliferation and differentiation of CD4^+^ T cell, attributed to its inhibitory effect on the expression of SOCS family proteins.^17^ Regulatory T cell (Treg), which are pivotal in modulating autoimmune responses, can become dysfunctional upon METTL3 deficiency, potentially leading to the onset of autoimmune disorders and immune escape.^18^ Collectively, these findings highlight the significant role of RNA m^6^A methylation in the development and homeostatic regulation of T cells.^19^

Although the KO of METTL3 or the application of METTL3 inhibitors in tumour cells has been shown to augment the efficacy of ICB therapy, there is a lack of attention to the impact of METTL3 on T cells within the tumour microenvironment. This oversight may stem from the potential disruption of T‐cell homeostasis by METTL3 deletion. Similarly, DNA methylation plays a crucial role in the normal development of thymocytes. It has been documented that the conditional KO of DNMT1 using Lck‐Cre leads to impaired thymocyte development and damaged T‐cell survival.^20^, ^21^ Conversely, the treatment of CAR‐T cells with low‐dose DNA methylation inhibitor decitabine has been shown to enhance anti‐tumour activity, cytokine production and proliferation in both in vitro and in vivo studies.^22^ Additionally, decitabine has been demonstrated to potentiate the anti‐tumour effect of anti‐PD‐1 therapy by preventing the depletion of CD8^+^ T cells.^23^ These findings suggest that KO and pharmacological inhibition can exhibit distinct effects, possibly attributable to the dose‐dependent nature of the intervention.

In this study, we identify that METTL3 functions as an immune suppressor that limits anti‐tumour immunity in melanoma and colon cancer. We discovered METTL3 inhibition with STM2457 treatment enhances tumour killing by promoting T‐cell function and further improves anti‐PD‐1 therapy in combination of STM2457. Data from single‐cell RNA sequencing (scRNA‐seq) suggest that METTL3 inhibition regulate CD8^+^ T cells to reduce exhaustion and enhance T‐cell activation. Mechanically, METTL3 inhibition triggers viral defense, interferon response and major histocompatibility complex (MHC)‐I expression, thereby inflaming immunologically cold tumours; and METTL3 inhibition sustains T‐cell persistence, simultaneously affecting both tumour cells and T cells to sensitise ICB‐based immunotherapy.

## 2 MATERIALS AND METHODS

### 2.1 Cell lines and cell culture condition

B16 cells THP‐1 cells were cultured in RPMI 1640 medium (Gibco) supplemented with 10% foetal bovine serum (FBS, Lonsera), 1% GlutaMAX (Gibco) and 1% non‐essential amino acids (NEAA, Gibco). MC38 cells, A375 cells and A1847 cells were cultured in HG DMEM medium (Gibco) supplemented with 10% FBS, 1% GlutaMAX and 1% NEAA. All the cell lines were tested negative for mycoplasma.

### 2.2 CRISPR‐Cas9‐mediated gene knockout

*Ythdf1*, *Ythdf2*, *Ythdf3* and *Mettl3* KO B16 cell were generated using the lentivirus‐mediated CRISPR‐Cas9 technology. The sgRNA oligos for target genes were annealed and cloned into the BsmB1 (Thermo Fisher Scientific) digested plasmid lentiCRISPR v2 vector (52961, Addgene). To KO target genes, B16 cells were transiently transfected with lentiCRISPR‐v2 vector carrying respective sgRNAs and selected with puromycin (puro; 1 µg/mL for B16 cells) at low density in six‐well plates for 5–7 days. Colonies were amplified and validated for KO by immunoblots.

### 2.3 Quantification of METTL3‐dependent m^6^A by LC‒MS

The total RNA was extracted from the cells with TRIZOL (MRC) reagent according to the manufacturer's instructions. Two hundred nanograms of extracted RNA from 5 µM STM2457‐treated B16 cells and THP‐1 was digested into nucleosides by nuclease P1 (1 U M0660S, NEB) and shrimp alkaline phosphatase (rSAP, 1 U, M0371S, NEB) in 25 µL RNase‐free water at 37°C overnight. The mixture was diluted to 50 µL, 5 µL of which was injected into an LC–MS/MS system consisting of a high‐performance liquid chromatographer (ExionLC AD) equipped with a ZORBAX SB‐Aq column (Agilent) and a Triple Quad 4500 (AB SCIEX) mass spectrometer in positive ion mode by multiple‐reaction monitoring. Mass transitions of m/z 268.0–136.0 (A), m/z 245.0–113.1 (U), m/z 244.0–112.1 (C), m/z 284.0–152.0 (G) and m/z 282.0–150.1 (m^6^A) were monitored and recorded. A concentration series of pure commercial nucleosides (MCE) was employed to generate standard curves. Concentrations of nucleosides in samples were obtained by fitting signal intensities to standard curves with certain ratios calculated subsequently. The concentrations of m^6^A and A were calculated by comparison with a standard curve, and their ratio was used to represent the m^6^A modification level.

### 2.4 Animal experiments

C57BL/6J, nude mouse or NCG mice (6‒8 weeks) were purchased from GemPharmatech. 1 × 10^5^ edited B16 or 4 × 10^5^ MC38 or 1 × 10^6^ A375 cells were suspended in 200 µL of PBS/Matrigel (Corning) (1:1) and then subcutaneously inoculated into flank of each mouse. Tumours were measured every 1–2 days once palpable and tumour surface was measured with a caliper using formula: *V* = 1/3 × *L* × *W* × *H*. Tumour bearing mice were divided randomly into four groups: (1) control, immunoglobulin (Ig)G1 Ab (BE0083, Bioxcell); (2) PD‐1 Ab (BE0146, Bioxcell); (3) STM2457 (DC53045, DC Chemicals); (4) combined therapy: PD‐1 Ab + STM2457. All treatments were conducted by intraperitoneal injection. PD‐1 Ab was given every 3 days with a dosage of 250 µg per mouse in B16 melanoma mouse model, once a week with a dosage of 100 µg per mouse in MC38 colon mouse model. The STM2457 treatment was given at a dosage of 50 mg/kg of mouse body weight every day. Pan T cells were generated from BALB/c mice and were injected into nude mice via the tail vein. All mouse procedures were performed in accordance with the institutional protocol guidelines. The mice were euthanised before the tumour reached a volume of 2000 mm^3^. The tumours were then excised, weighed using a balance, and photographed for documentation.

### 2.5 Tumour cell and T‐cell co‐culture assay

OT‐I CD8^+^ T cells were generated from OT‐I mice (Donated form Penghui Zhou Laboratory). In brief, spleens were harvested and cells were sorted with mouse CD8^+^ T‐cell isolation kit (19853, stem cell) or mouse pan T‐cell isolation kit (19851, stem cell). Human T cell were enriched from the peripheral blood mononucle (PBMC) from healthy donors using a ‘pan T‐cell isolation kit’ (130‐096‐535, Miltenyi Biotec). T cells were maintained in media (RPMI‐1640, 10% FBS, 1× GlutaMAX, 1× NEAA, 2‐mercaptoethanol) supplemented with 100 ng/mL mouse/human IL‐2 (Peprotech) and activated by mouse CD3/CD28 (11456D, Gibco) or human CD3/CD28 (11161D, Gibco) for 48 h and then expanded in IL‐2 alone or with 2 µM STM2457 for a further 48 h. Then, 5 µM STM2457 pre‐treated T cell and B16 cells expressing chicken ovalbumin (B16‐OVA) and Luciferase reporter were co‐cultured as shown in Figure 4D. Target cell viability was monitored 48 h later by adding 100 µL/well of the substrate D‐luciferin (sodium salt) (Yeasen) at 150 µg/mL, then the viability of the target cells was monitored by a microplate reader.

### 2.6 Western blot

Western blots were performed using typical laboratory procedures. PVDF membranes were blocked using 5% non‐fat milk and were incubated with primary antibodies overnight at 4°C, antibodies: YTHDF1 (17479‐1‐AP, Proteintech); YTHDF2 (ARP67917_P050, Vivasysbio); YTHDF3 (25537‐1‐AP, Proteintech); and METTL3 (15073‐1‐AP, Proteintech). After washing with TBS supplemented with 1% Tween‐20, membranes were incubated with horseradish peroxidase (HRP)‐conjugated polyclonal goat anti‐rabbit or goat anti‐mouse secondary antibodies at room temperature for 1 h. ECL kit (P10300, NCM) was used to develop the signal. Bio‐Rad machine was employed to acquire band images. Antibodies used in this study are listed in Appendix.

### 2.7 Flow cytometry

Tumours were washed with PBS, discarded the necrotic parts of the tissue, cut into pieces <1 mm^3^, and put into 1.5 mL of preheated DMEM culture medium, with a final concentration of 1–2 mg/mL collagenase P (100×), 20 µg/mL DNase I enzyme (500×), digested at 37°C for 15 min, vibrated and pipetted, filtered with a 70 µm filter membrane, centrifuged to remove the supernatant, added 700 µL of lysis red reagent, lysed at 4°C for 10 min, then added an equal volume of PDS serum to terminate. Cells were re‐suspended with 100 µL PBS with 1 µL of FCblock (or 1% bovine serum albumin (BSA) + 2% FBS) for 15 min, and stained with the indicated antibodies. Antibodies: FITC anti‐mouse CD45 (147709, Biolegend); PE anti‐mouse CD8 (100707, Biolegend); APC anti‐mouse CD4 (100411, Biolegend); FITC anti‐mouse CD8 (100803, Biolegend); PE anti‐human CD366 (Tim‐3) (364805, Biolegend); APC anti‐human CD279 (PD‐1) (379207, Biolegend); APC anti‐mouse IFNγ (50709‐R348‐A, Sino Biological); PE anti‐human/mouse granzyme B (GzmB; 372207, Biolegend). Flow cytometry was performed on CytoFLEX cytometer (BD Biosciences). Data were analysed using FlowJo software (FlowJo).

### 2.8 Cell viability assay

Cell viability was assessed using Cell Counting Kit‐8 Assay (C0037, Beyotime) following the manufacturer's instructions. The cells were exposed to different concentrations of STM2457 (0, 1, 5 and 10 µM) in 96‐well plates for 3 days. A microplate reader was used to measure the absorbance of the samples.

### 2.9 Immunohistochemistry

For immunohistochemistry analysis, tumour tissues from mice were fixed in 4% paraformaldehyde fix solution (4% PFA) at 4°C overnight, paraffin‐embedded and sectioned and then mounted. Sections were deparaffinised in xylene, rehydrated and washed in PBS. Subsequently, sections were boiled with antigen unmasking solution (H‐3300, Vector Labs) for 20 min, blocked with 10% normal goat serum in PBS at room temperature for 1 h, and stained by standard procedures using antibodies against CD8 (Cell Signaling Technology, 98941T). Then, the sections were washed by PBST for five times, incubated with HRP goat anti‐rabbit IgG at 25°C for 1 h and treated with diaminobenzidine (DAB) substrate kit (P0202, Beyotime) for 5 min and then counterstained with haematoxylin. Finally, all the mouse and human colon tissue slides were imaged.

### 2.10 Total RNA extraction and qRT‐PCR

Total RNA from cell lines was isolated using TRIZOL (MRC) method. RNA quantity and quality were assessed with NanoDrop One C spectrophotometer (ND‐ONEC‐W, Thermo Fisher Scientific). Only RNA with an absorbance read ratio 260/280 between 1.8 and 2.0 was used for experiments. Then, 1 µg of total RNA was used for cDNA synthesis with HiScript II Q RT SuperMix for qPCR (R222‐01, Vazyme), and the diluted cDNA was used as template for qPCR with ChamQ SYBR qPCR Master Mix (Q311‐02, Vazyme). Specific primers were designed for each gene transcript and are listed in Appendix: Reagents.

### 2.11 mRNA stability measurements

B16 cells with KO of *Ythdf2*, *Mettl3* and control or treatment with STM2457 were treated with 100 nm actinomycin for 0, 4 and 8 h, respectively, and then these cells were collected. Subsequently, mRNA levels were quantified by RT‐qPCR with gene‐specific qPCR primers.

### 2.12 RNA sequencing

#### 2.12.1 Total RNA‐seq construction and RNA‐seq analysis

Sequencing libraries were prepared according to the VAHTS Universal RNAseq Libraries Prep Kit (NR605, Vazyme). Modified and purified cDNA library were undergone high throughput sequencing using Illumina HiSeq platform with PE150. Raw reads were trimmed with TrimGalore (version 0.6.4) (https://github.com/ FelixKrueger/TrimGalore), then the cleaned reads were aligned to the mouse Gencode (vM15) transcriptome with RSEM (version 1.2.22).^24^ The gene expression were normalised by DESeq2 (version 1.30.0).^25^ Differentially expressed genes (DEGs) were obtained using DESeq2 (version 1.30.0).^25^ Gene ontology (GO) analysis was performed using clusterProfiler (version 3.18.0).^26^

#### 2.12.2 scRNA‐seq construction and analysis

Single‐cell suspensions from each sample were loaded onto 10× Genomics Chromium v3.1 system to generate single‐cell gel beads‐in‐emulsion (GEMs), where all generated full‐length cDNA share a common 10× barcode. After incubation, GEMs were disrupted and cDNA was amplified via PCR. The single‐cell 3′ gene expression libraries were constructed using 10 µL (a proportion of 25%) of the total cDNA and purified with SPRIselect. Libraries were quality controlled by Qsep100 for sized distribution and by Qubit 4.0 fluorometer for concentration quantification. Finally, sequencing was performed on Illumina NovaSeq system with 150 G paired bases in PE150 mode.

For scRNA‐seq data analysis, raw reads were trimmed with TrimGalore (version 0.6.4) (https://github.com/FelixKrueger/TrimGalore), then the cleaned reads were aligned to mouse mm10 genome by STARsolo (version 2.7.8a) with the setting ‘–outSAMattributes NH HI AS nM CR CY UR UY –readFilesCommand zcat –outFilterMultimapNmax 100 –winAnchorMultimapNmax 100 –out‐MultimapperOrder Random –runRNGseed 777 –outSAMmultNmax 1’, then gene quantification was performed by scTE pipeline as previously described.^27^, ^28^ The count matrix was normalised using NormaliseData function of Seurat (version 4.0.0), and the top 2000 most highly variable genes were used for PCA, and the first 20 principle components were used.^29^ To integrate cells into a shared space from different datasets for unsupervised clustering, we used the Seurat to do batch effect correction. The differential expressed genes were obtained using the FindAllMarkers function Seurat with the ‘logfc.threshold = .25, test.use = “wilcox,” min.pct = .3, only.pos = T, min.diff.pct = .1’ setting. Other analysis was performed by SCANPY.^30^

### 2.13 Quantification and statistical analysis

Data are presented as mean ± SEM or mean ± standard deviation as indicated in the figure legends. Unpaired two‐tailed Student's *t*‐test, two‐way analysis of variance (ANOVA) with Sidak's multiple comparisons test were used to assess statistical significance. The *p*‐value, *t*‐ratio were calculated with the Prism 8 software. A *p*‐value <.05 was considered as being statistically significant, ^*^ *p* < .05, ^**^ *p* < .01 and ^***^ *p* < .001. No statistical method was used to predetermine sample size.

## 3 RESULTS

### 3.1 METTL3 inhibition induces an immune response that hinders tumour growth

**FIGURE 1:**
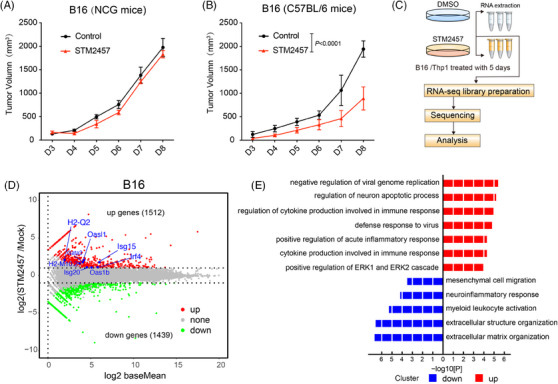
STM2457 killing tumour effect dependents on immune system. (A and B) Tumour growth curves of B16 tumour engraftment NCG and C57BL/6 mice treated with STM2457 (*n* = 5; error bar, SEM; two‐way analysis of variance [ANOVA]). (C) Diagram showing the process of RNA sequencing (RNA‐seq) and analysis in B16 cells treated with 5 µM STM2457 versus DMSO. (D) Volcano plot showing differentially expressed genes (DEGs) from RNA‐seq results of B16 cells treated as in (C). Red dots represent upregulated genes and green dots represent downregulated genes. (E) Gene ontology (GO) analysis of DEGs in B16 cells treated with STM2457 versus DMSO.

To investigate the functional role of METTL3 in tumour growth, several cancer cell lines were treated with METTL3 inhibitor STM2457. Consistent with previous studies, STM2457 significantly inhibited the proliferation of acute myeloid leukaemia cells, such as THP‐1 cells^31^ (Figure S1A). Additionally, a mild growth inhibition was observed in B16 and MC38 cells (Figure S1A). LC‒MS/MS assays showed that STM2457 treatment significantly decreased m^6^A mRNA levels (Figure S1B). To elucidate the functional role of METTL3 in tumour growth in vivo, B16 cells were inoculated into immunodeficient NOD CRISPR Prkdc Il2r gamma (NCG) mice. Surprisingly, there were no significant differences in tumour growth between the STM2457 treatment groups and the control group (Figure 1A). These data collectively suggest that METTL3 inhibition has little effect on B16 melanoma cell proliferation in vitro and tumour growth in immunodeficient hosts in vivo. Next, to assess the impact of an intact immune system, we inoculated B16 cells into immunocompetent C57BL/6 mice and administered STM2457. Notably, STM2457 therapy significantly hindered tumour progression (Figure 1B). These results indicate that the anti‐tumour efficacy of METTL3 inhibition depends on an intact immune system.

To further elucidate the molecular mechanism of targeting METTL3 on tumour growth inhibition, we treated B16 cells with STM2457 for 5 days. Subsequently, RNA‐seq was performed to identify DEGs (Figure 1C,D). Of note, GO analysis indicated that genes associated with anti‐viral responses and interferon signalling pathways were significantly upregulated following METTL3 inhibition (Figure 1E). Importantly, the expression of type I interferons and interferon‐stimulated genes (ISGs) was significantly increased after METTL3 inhibition (Figures 1D and S1E). Comparable activation of the transcriptome was observed in THP‐1 cells following STM2457 treatment (Figure S1C,D,F). In summary, these results suggest that METTL3 inhibition can induce potent anti‐tumour immunity.

### 3.2 METTL3 inhibition enhances the efficacy of anti‐PD‐1 therapy

**FIGURE 2:**
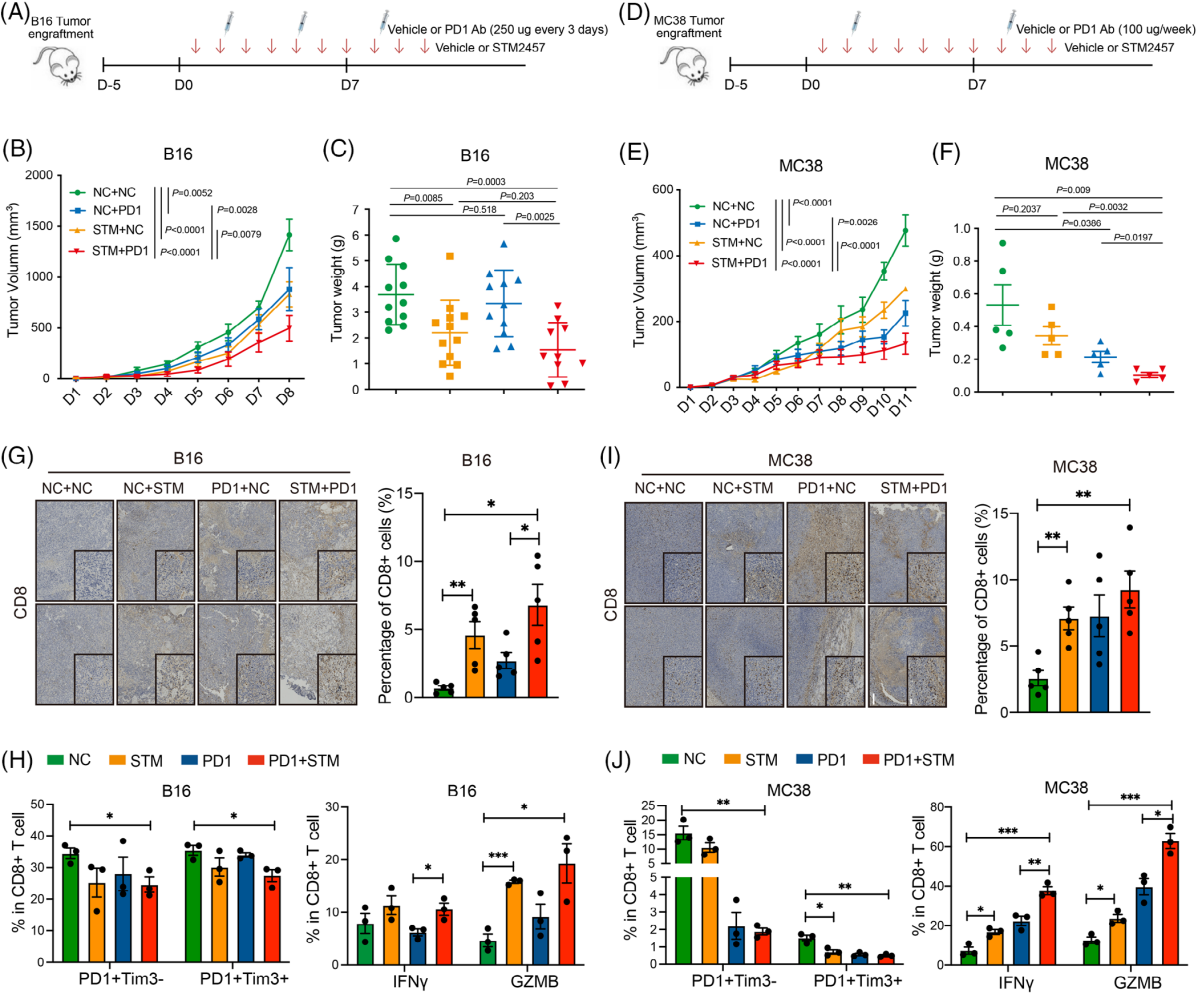
METTL3 inhibition sensitises tumours to anti‐PD‐1 immunotherapy. (A and D) Diagram of the experimental design. Mice were assigned to four groups: vehicle, PD‐1 Ab, STM2457 or PD‐1 Ab + STM2457 after B16 (A) and MC38 (D) tumour engraftment. Vehicle or PD‐1 Ab was administered intraperitoneally every 3 days for B16, and once a week for MC38. (B and E) Tumour growth curves of B16 (B) and MC38 (E) tumour engraftment C57BL/6 mice treatment by PD‐1 Ab in combination with STM2457 (B: *n* = 11; error bar, SEM; two‐way analysis of variance [ANOVA]; E: *n* = 5; error bar, SEM; two‐way ANOVA). (C and F) Tumour weight of C57BL/6 mice after treated as described in (A) and (D) (C: *n* = 11 of two biological replicates, SEM; unpaired *t*‐test; F: *n* = 5 of two biological replicates, SEM; unpaired *t*‐test). (G and I) Immunohistochemistry staining (left) and quantitative data (right) of CD8 staining in B16 (G) and MC38 (I) tumour sections. Tumours were treated as described in (A) and (D). Low magnification (scale bar = 200 µm); inset image: high magnification (scale bar = 50 µm) (*n* = 3, error bar, SEM; unpaired *t*‐test, ^*^ *p* < .05, ^**^ *p* < .01, ^***^ *p* < .001). (H) Flow cytometry showing the ratio of PD1+Tim3‒ and PD1+Tim3+ cells in CD8^+^ T cells in B16 tumours (left); the ratio of interferon‐gamma (IFNγ) and granzyme B (GzmB)‐positive cells in CD8^+^ T cells in B16 tumours (right). Tumours were treated as described in (A) (*n* = 3, error bar, SEM; unpaired *t*‐test, ^*^ *p* < .05, ^**^ *p* < .01, ^***^ *p* < .001). (J) Flow cytometry showing the ratio of PD1+Tim3‒ and PD1+Tim3+ cells in CD8^+^ T cells in MC38 tumours (left); the ratio of IFNγ and GzmB‐positive cells in CD8^+^ T cells in MC38 tumours (right) (*n* = 3, error bar, SEM; unpaired *t*‐test, ^*^ *p* < .05, ^**^ *p* < .01, ^***^ *p* < .001).

Previous research has shown that epigenetic inhibitors, such as DNA methyltransferase inhibitors (DNMTi) and histone deacetylase inhibitors, can activate interferon pathway‐associated immune genes, thereby reversing the effect of ICB.^6^, ^32^ The inhibiting of METTL3, an RNA m^6^A methyltransferase, has been shown to trigger viral defense mechanisms and interferon responses within B16 melanoma cells (Figure 1D,E). Additionally, we observed an upregulation in the expression of specific MHC‐I‐related genes, including H2‐Q2 and H2‐M10.2, indicating a potential synergistic effect with ICB treatment (Figure 1D). To assess the therapeutic potential of METTL3 inhibition in ICB therapy, we administered a PD‐1 monoclonal antibody (anti‐PD‐1) and STM2457 to immunocompetent C57BL/6 mice bearing B16 melanoma cells (Figure 2A).

As expected, we observed a modest therapeutic effect with either anti‐PD‐1 treatment alone or STM2457 treatment alone compared to the control group of mice with B16 tumours (Figures 2B,C and S2A). In contrast, the combination therapy of STM2457 with anti‐PD‐1 exhibited significant tumour‐suppressive effects compared to single‐agent therapy alone (Figures 2B,C and S2A). Particularly, in the anti‐PD‐1 sensitive MC38 colon tumour model, the combination therapy further enhanced the tumour‐suppressive effects (Figure 2D‒F). Given the crucial role of CD8^+^ T cells in anti‐tumour responses, we performed immunohistochemical staining of tumour tissues with a CD8‐specific antibody. This analysis revealed that METTL3 inhibition enhances the infiltration of CD8^+^ T cells (Figure 2G). Subsequent flow cytometry analysis revealed that the combination therapy of STM2457 with anti‐PD‐1 reduced CD8^+^ T‐cell exhaustion in B16 tumours and significantly enhanced CD8^+^ T‐cell cytotoxicity by upregulating the expression of IFNγ and GzmB (Figure 2H). Similar observations were made in MC38 tumours (Figure 2I,J). Notably, STM2457 monotherapy was able to inhibit the growth of human melanoma A375 tumours both in vivo and in vitro (Figures S1A and S2C‒E). Taken together, these results suggest that targeting METTL3 represents a promising therapeutic strategy for enhancing the efficacy of anti‐tumour immunotherapy.

### 3.3 METTL3 inhibition improves anti‐PD‐1 therapy through an m^6^A‐YTHDF2‐dependent manner

The role of RNA m^6^A in regulating gene expression is mostly mediated by readers, the YTH domain‐containing family proteins.^33^, ^34^ To investigate the effects of METTL3 on tumour immunity, we KO the m^6^A reader *Ythdf1‐3* and *Mettl3* in B16 cells using CRISPR‐Cas9 (Figure S3A). Interestingly, the KO of *Ythdf1‐3* had no effect on cell proliferation, whereas the KO of METTL3 markedly impeded it, contrasting with the effects of METTL3 inhibition by STM2875 treatment (Figure S1B, S3B). We further overexpressed wild‐type (WT) or catalytically dead mutants (DPPW 395–398 APPA) of *Mettl3* and found that only WT *Mettl3* could rescue the proliferation inhibition (Figure S3C). These findings suggest that the inhibition of METTL3 does not have the same effects as complete *Mettl3* KO in terms on cellular proliferation in vitro, possibly due to dosage effects.

**FIGURE 3:**
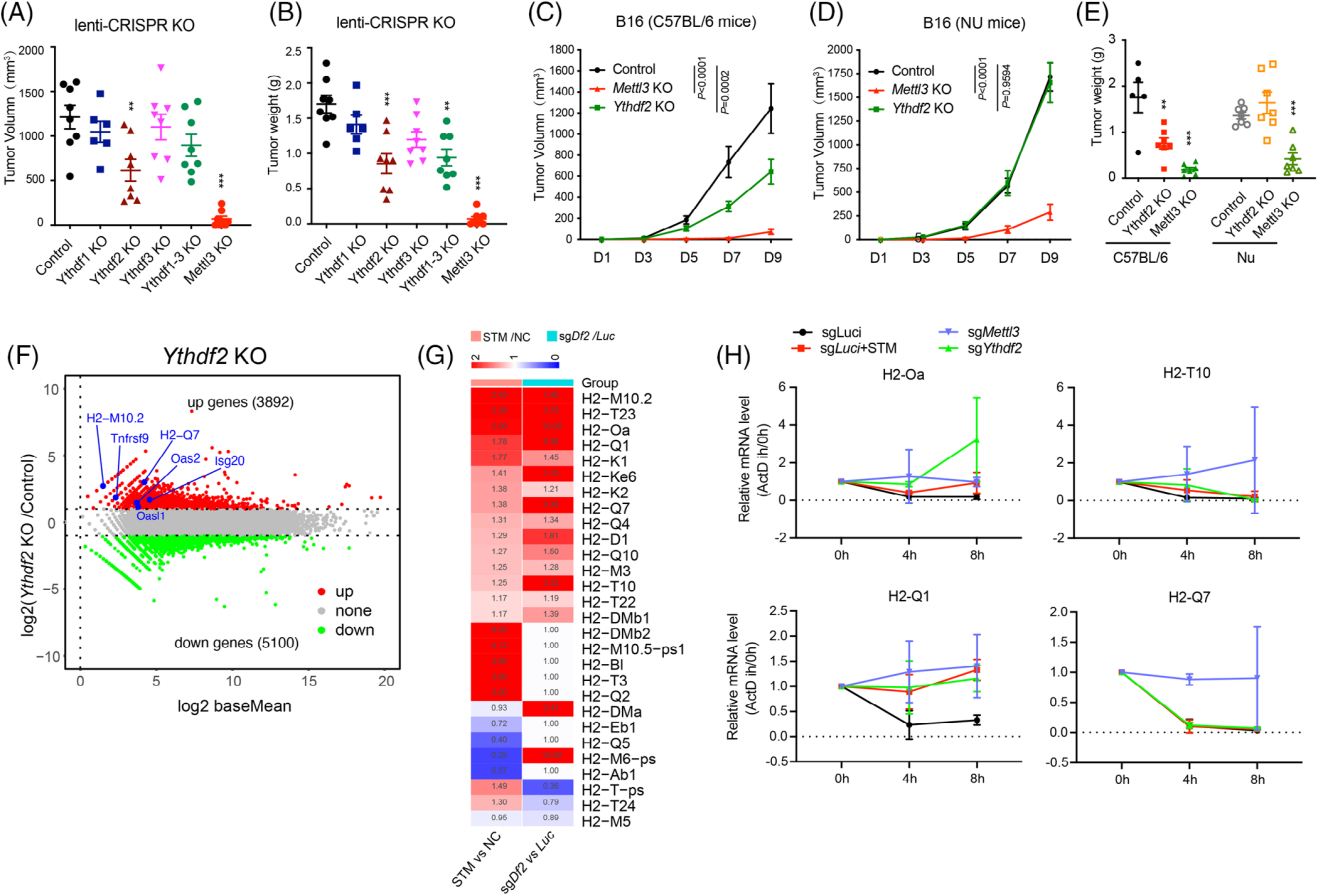
YTHDF2 acts as the downstream reader of METTL3 inhibition. (A and B) Tumour volume (A) and weight (B) of in C57BL/6 mice injected with B16 cells that were genetically modified by CRISPR/Cas9 to knockout Ythdf1‐3, Mettl3 or luciferase (as a control) genes (*n* = 8, error bar, SEM; unpaired *t*‐test, ^*^ *p* < .05, ^**^ *p* < .01, ^***^ *p* < .001). (C and D) Tumour growth curves of B16 tumours in C57BL/6 (C) and nude (D) mice, B16 cells were genetically modified by CRISPR/Cas9 to knockout Ythdf2, Mettl3 or luciferase (as a control) genes (*n* = 7, error bar, SEM; two‐way analysis of variance [ANOVA]). (E) Tumour weight of C57 and nude mice subcutaneously injected with B16 cells that were genetically modified by CRISPR/Cas9 to knockout Ythdf2, Mettl3 or luciferase (as a control) genes (for control group: *n* = 5, for test group: *n* > 5, error bar, SEM; unpaired *t*‐test, ^*^ *p* < .05, ^**^ *p* < .01, ^***^ *p* < .001). (F) Volcano plot showing differentially expressed gene (DEG) results of Ythdf2 knockout B16 cells. Red dots represent upregulated genes and green dots represent downregulated genes. (G) Heatmap depicting differential expression of MHC‐I genes in B16 cells. Comparison shown between STM‐treated versus control (NC) cells, and Ythdf2 knockout (sgYthdf2) versus control (sgLuciferase) cells. (H) mRNA lifetime of selected genes in Mettl3 knockout, Ythdf2 knockout, STM treated and control cells, measured by RT‐qPCR. Cells were treated with actinomycin D (ActD) and samples were collected at 0, 4 and 8 h (*n* = 2).

We then inoculated B16 cells into immunocompetent C57BL/6 mice and observed that the KO of *Ythdf1/3* alone did not affect tumour growth compared to the control group (Figures 3A,B and S3D). However, in contrast to control counterparts, *Ythdf2*/*Mettl3*‐KO tumour cells were notably rejected in immunocompetent mice (Figure 3C). Subsequently, to evaluate the role of an intact immune system, we inoculated *Ythdf2/Mettl3* KO B16 cells into the T‐cell‐deficient nude mice. Our findings revealed that the KO of *Ythdf2* had minimal effects on B16 melanoma tumour growth in immunodeficient hosts, suggesting that the anti‐tumour effect of Ythdf2 KO is T‐cell‐dependent (Figures 3D and S3E). Concurrently, *Mettl3* KO‐inhibited tumour growth in both immunocompetent and immunodeficient mice, confirming that the effects of inhibiting METTL3 and complete KO are different (Figure 3C‒E). It is worth mentioning that the KO of *Ythdf2*/*Mettl3* in B16 cells did not affect T‐cell exhaustion (Figure S3F). Additionally, the ISGs such as *Isg20*, *Oasl1* and some MHC‐I genes such as *H2‐M10.2* were upregulated upon *Ythdf2* deletion in B16 cells, and upregulated genes of T‐cell activation pathway were enriched (Figures 3F and S3G). Given that the function of *Ythdf2* is to accelerate mRNA decay, we performed RNA lifetime profiling of *Ythdf2*‐KO, *Mettl3*‐KO, STM2457 treatment, and control samples treated with actinomycin D by qRT‐PCR. Of note, the intervention of *Mettl3*/*Ythdf2* prolongs the lifespan of MHC‐I mRNAs that upregulated upon both STM2457 treatment and *Ythdf2* deficiency (Figure 3G,H). Taken together, these findings suggest that METTL3 and YTHDF2 are both crucial for anti‐tumour immunity, and YTHDF2 probably functions as a downstream executed reader of the tumour‐killing effect of STM2457 treatment.

### 3.4 Sampling cell fates of tumour microenvironment with STM2457 treatment by scRNA‐seq

**FIGURE 4:**
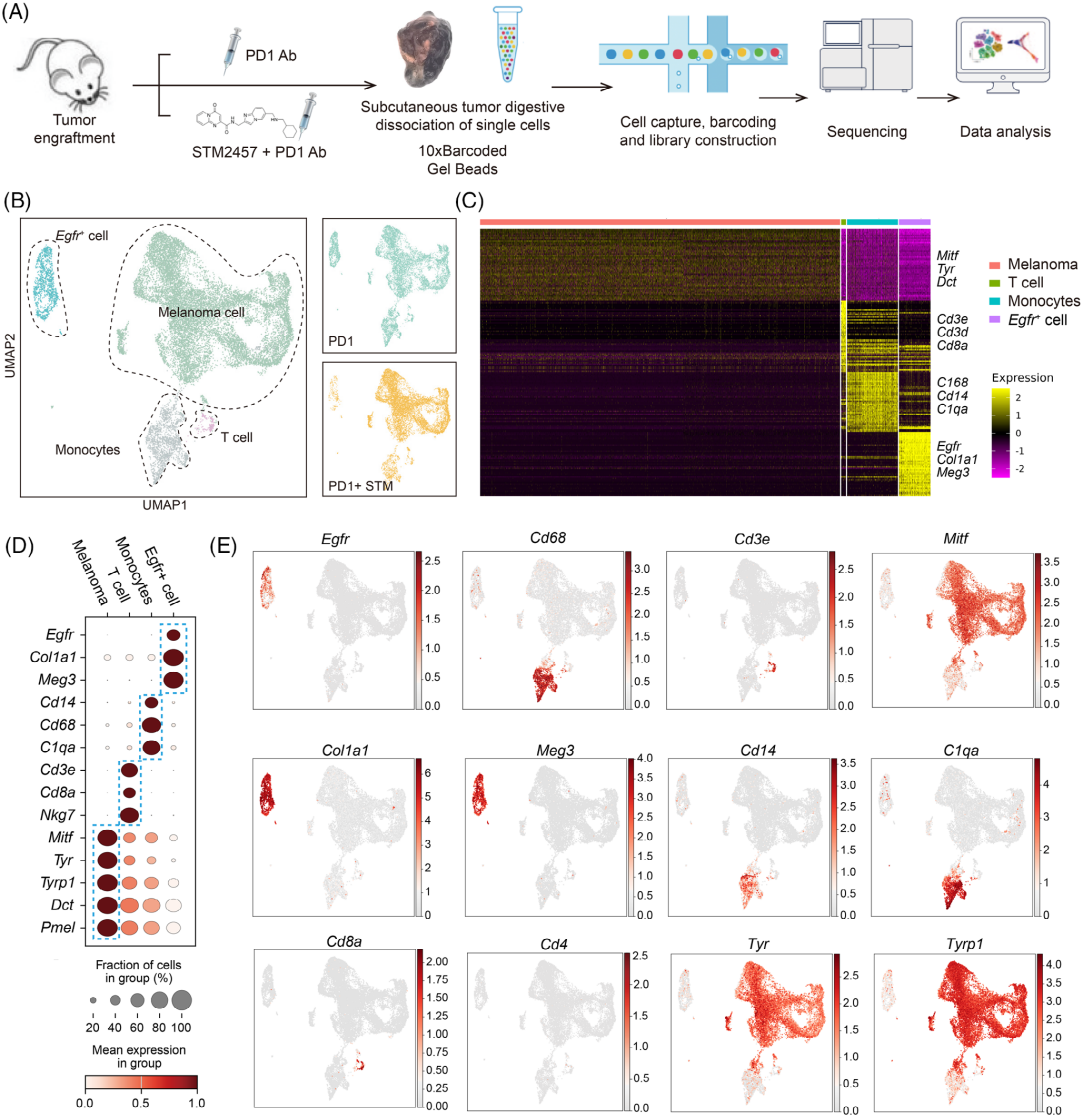
Sampling cell fates of tumour microenvironment with STM2457 treatment by single‐cell RNA sequencing (scRNA‐seq). (A) Diagram of the experimental design. Tumour tissue is digested to prepare a single‐cell suspension and subjected to 10× scRNA‐seq construction and analysis. (B) Uniform Manifold Approximation and Projection (UMAP) layout showing clusters of single cells separated from tumours. Each dot represents one cell, with colour code for cell type (left). Differences in cellular composition among PD‐1 and PD‐1 + STM (right). (C) Heatmap depicting the marker genes expressed across different cell types. (D) Dotplot showing the average expression levels per cluster of the differentially expressed markers in each cluster. (E) UMAP plots showing the expression of maker genes for particular cell types. Gene expression levels are indicated by shades of red.

As described above, the anti‐tumour efficacy of METTL3 inhibition is dependent on an intact immune system, especially T cells. To better understand the effect of STM2457 in tumour microenvironment when combined with anti‐PD‐1 treatment, we conducted scRNA‐seq on tumour tissues treated with either anti‐PD‐1 alone or the combination of STM2457 and anti‐PD‐1 utilising the 10× Genomics platform (Figure 4A). In total, we analysed 18 341 single cells after rigorous quality control, with each cell detecting approximately 4000 expressed genes (Figure S4A‒D). Uniform Manifold Approximation and Projection analysis yielded well segregated clusters for different cell types and most cell types were conserved between the anti‐PD‐1 and anti‐PD‐1 + STM2457 groups, including melanoma cell, monocytes and T cells (Figure 4B). Among them, we labelled melanoma with *Mitf*, *Tyr*, *Tyrp1*, *Dct* and *Pmel*; monocytes with *Cd14*, *Cd68* and *C1qa*; T cell with *Cd3e*, *Cd8a* and *Nkg7*; and a group of Egfr+ cells expressing *Egfr* (Figures 4B‒E and S4E). Notably, the majority of CD3^+^ T cells are predominantly CD8^+^ rather than CD4^+^ cells (Figures 4E and S4F).

### 3.5 METTL3 inhibition improves T‐cell persistence in ICB treatment

**FIGURE 5:**
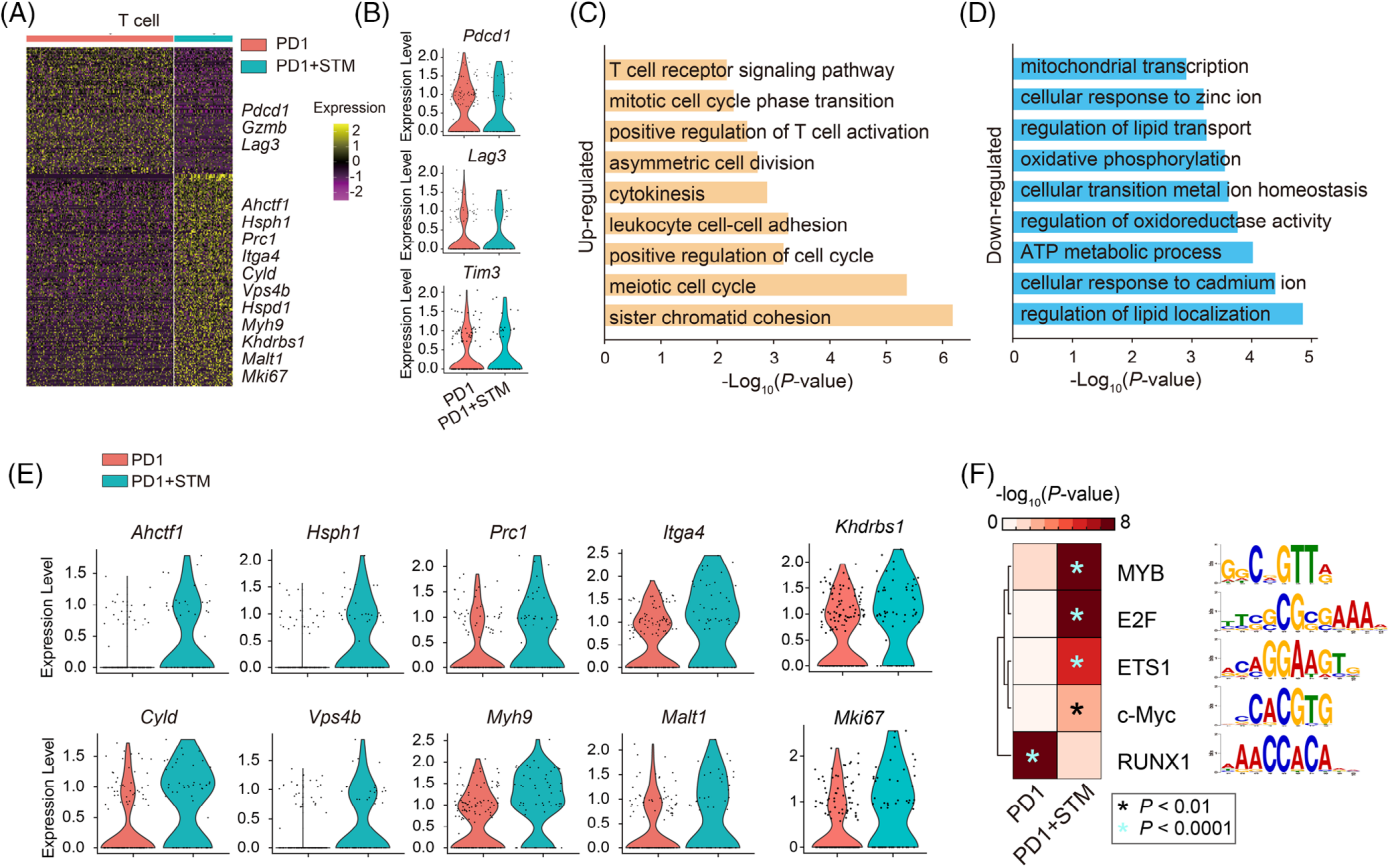
METTL3 inhibition improves T‐cell persistence in immune checkpoint blockade (ICB) treatment. (A) Heatmap depicting the differential expressed genes between control (NC) and STM treatment in T cells. Selected genes are labelled in the right. (B) Violin plots showing the differential expression of T‐cell exhaustion‐related genes upon STM treatment. (C and D) Gene ontology (GO) analysis for the differential expressed genes from panel (E). (E) Violin plots showing the differential expression of T‐cell activation‐related genes upon STM treatment. (F) Heatmap showing the enrichment for potential transcription factor (TF) motifs.

As the main force in the immune system's tumour‐killing activity, CD8^+^ T cells may play a crucial role in enhancing the tumour‐killing effect with METTL3 inhibition. To investigate it, we compared the gene expression of CD8^+^ T cells between anti‐PD‐1 and anti‐PD‐1 + STM2457 by scRNA‐seq data (Figure 5A). Notably, the T‐cell exhaustion markers *Pcdc1*, *Lag3* and *Tim3* had decreased expression with STM2457 treatment in the scRNA‐seq analysis (Figure 5A,B). Further GO analysis showed that the upregulated genes are tightly associated with meiotic cell cycle and T‐cell activation, suggesting that METTL3 inhibition promotes T‐cell proliferation and activation. Indeed, T‐cell activation associated genes, including *Hsph1*, *Itga4*, *Cyld*, *Vps4b*, *Myh9* and *Malt1*, were significantly upregulated in the anti‐PD‐1 + STM2457 group (Figure 5C‐E).^35^, ^36^ Meanwhile, the downregulated genes are involved in mitochondrial metabolism and oxidative phosphorylation (Figure 5D), which appears contradictory to the existing literature suggesting that CD8^+^ cells require active mitochondrial metabolism.^37^, ^38^ This discrepancy may deserve further investigation. To elucidate the underlying molecular mechanisms responsible for the enhanced T‐cell persistence observed in ICB treatment with STM2457, we performed an enrichment analysis to identify potential upstream transcription factors (TFs) based on the DEGs between the two treatment groups. Notably, we observed a significant enrichment of motifs corresponding to MYB, E2F, ETS1, and the c‐Myc family in the anti‐PD‐1 + STM2457 group (Figure 5F). Conversely, the RUNX1 family motif was specifically enriched in the single anti‐PD‐1 group (Figure 5F). Previous studies have established that RUNX1 is a crucial TF involved in regulating exhausted T cell.^39^, ^40^ On the other hand, MYB, E2F and c‐Myc have been associated with promoting proliferation and stem cell‐like characteristics,^41^, ^42^, ^43^ while ETS1 is known to regulate naive T cell.^39^, ^40^ Taken together, these results suggest that T cells in the STM2457 treated tumour microenvironment undergo significant cell proliferation and activation, indicating that METTL3 inhibition potentiate anti‐PD‐1 therapy by improving T‐cell persistence.

### 3.6 METTL3 inhibition enhances T‐cell killing in vitro and in vivo

**FIGURE 6:**
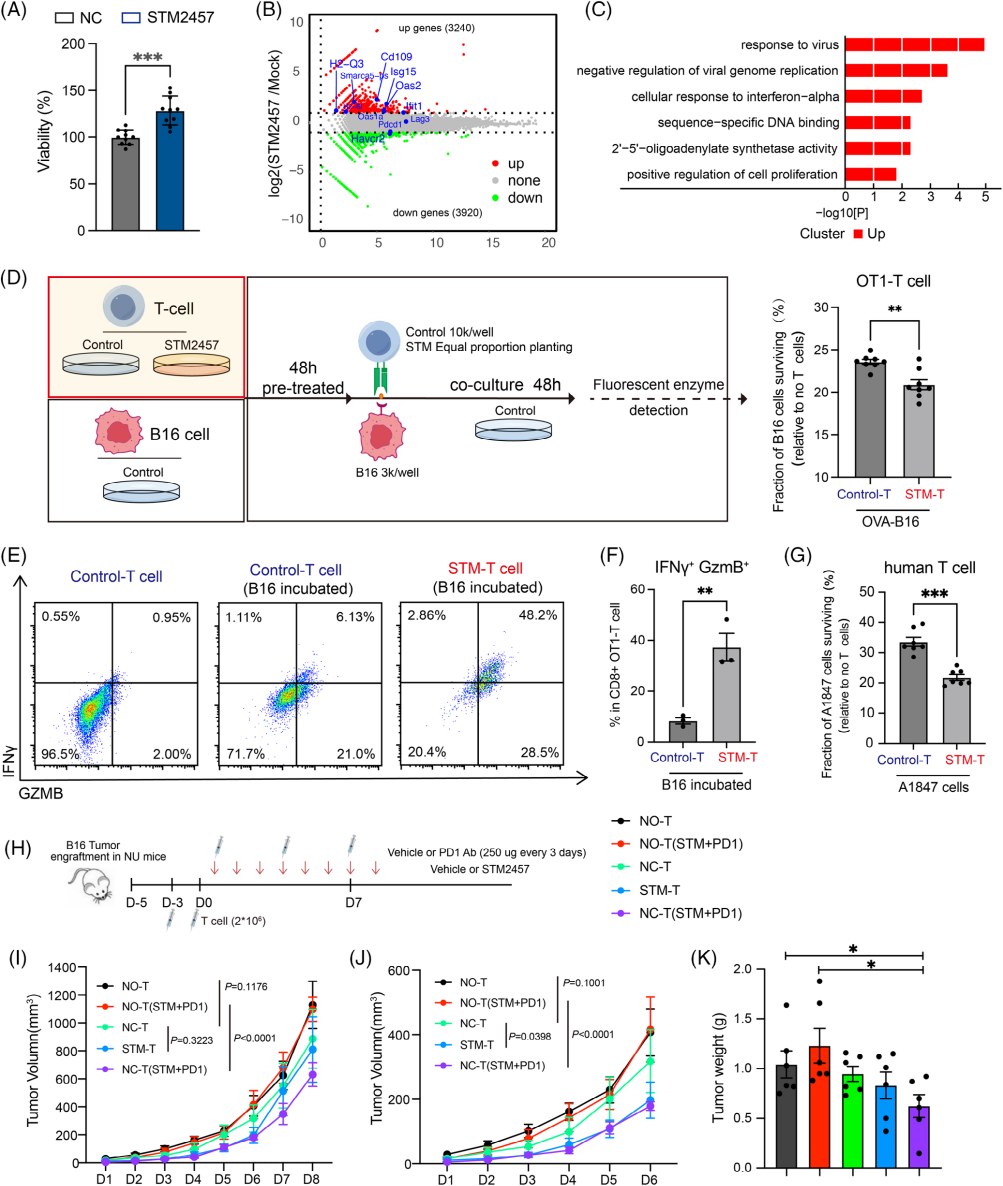
METTL3 inhibition promotes T‐cell proliferation and augments tumour killing in vitro and in vivo. (A) Cell viability assay showing percentage of pan T‐cell viability in control (NC) and STM2457 treatment conditions (*n* = 10 technical replicates with at least four biological replicates; error bar, SEM; ^***^ *p* < .001). (B) Volcano plot showing differentially expressed genes (DEGs) from RNA sequencing (RNA‐seq) results of pan T cells. Red dots represent upregulated genes and green dots represent downregulated genes. (C) Gene ontology (GO) analysis of DEGs in T cells treated with STM2457 versus DMSO. (D) Diagram depicting the co‐culture of pre‐treated OT‐I CD8^+^ T cells with untreated OVA‐B16 cells, the target cell viability was measured by fluorescent enzyme assay. Correspond result was showed on the right (*n* = 5 technical replicates with two biological replicates; error bar, SEM; ^**^ *p* < .01). (E) Flow cytometry detection of interferon‐gamma (IFNγ) and granzyme B (GzmB) expression of OT‐I CD8^+^ T cells in three groups: control T cells, control T cells incubated with B16 cells and STM2457 pre‐treated T cells incubated with B16 cells. (F) Flow cytometry analysis showing the ratio of OT‐I CD8^+^ T cells that are IFNγ+ and GzmB+ in two conditions: control T cells and STM2457 pre‐treated T cell, both incubated with B16 cells (*n* = 3, error bar, SEM; unpaired *t*‐test, ^*^ *p* < .05, ^**^ *p* < .01, ^***^ *p* < .001). (G) STM2457 pre‐treated human T cell (10k cells) cytotoxicity assay with A1847 cells (2k cells) (*n* = 6 technical replicates with three biological replicates; error bar, SEM; ^***^ *p* < .001). (H) Diagram of the experimental design. Mice were assigned to five groups: no T cell, no T cells with PD‐1 Ab + STM2457; T cell injected; STM2457 pre‐treated T cells or T cells injected with PD‐1 Ab + STM2457. (I and J) Tumour growth curves of B16 tumour engraftment nude mice with T cell injected and treatment by PD‐1 Ab in combination with STM2457 ended in day 8 (I) or day 6 (J) (*n* = 6; error bar, SEM; two‐way analysis of variance [ANOVA]). (K) Tumour weight of nude mice after treated as described in (H) (*n* = 6; error bar, SEM; unpaired *t*‐test).

ScRNA‐seq data revealed that the combination of STM2457 with ICB therapy effectively promotes T‐cell proliferation and activation. To explore this effect, T cells were isolated and cultured in vitro with STM2457 for 2 days. The results revealed an increase in T‐cell proliferation and a decrease in the expression of exhaustion‐associated genes (i.e., *Tim3*, *Pdcd1* and *Lag3*) upon STM2457 treatment (Figures 6A and S5A). We further conducted RNA‐seq analysis to provide insights into the molecular changes induced by STM2457, which indeed showed downregulation of T‐cell exhaustion makers such as *Pdcd1* and *Tim3*, along with activation of type‐1 interferon‐related genes and cell proliferation‐associated genes (Figures 6B,C and S5B,C). Thus, the above results demonstrate that STM2457 treatment can enhance T‐cell proliferation and activation by directly stimulating T cells.

To assess the impact of METTL3 inhibition on T‐cell killing capacity, we established an OT‐I CD8^+^ T‐cell cytotoxicity assay using OVA‐expressing B16 cells in vitro. T cells were pre‐treated with STM2457 and then co‐cultured them with OVA‐overexpressing (OVA‐OE) luciferase reporter B16 cells as shown in the diagram (Figure 6D). Notably, METTL3 inhibition significantly increased the cytotoxicity of T cells towards tumour cells (Figure 6D). This was further confirmed by flow cytometric analysis, which showed enhanced cytotoxicity of CD8^+^ T cells, accompanied by increased expression of IFNγ and GzmB (Figure 6E,F). Additionally, METTL3 inhibition was found to enhance the killing ability of human T cells in vitro, suggesting a promising role for METTL3 in human anti‐tumour immune responses (Figure 6G). Furthermore, we conducted an in vivo experiment to assess the effect of STM2457 on enhancing T‐cell‐mediated tumour elimination using T‐cell‐deficient nude mice with T‐cell injection (Figure 6H). Notably, the combination of STM2457 with anti‐PD‐1 therapy only exhibited tumour‐suppressive effects in nude mice in the presence of T‐cell injection (Figures 6I‒K and S5D). The injection of STM2457 pre‐treated T cell showed an enhanced tumour‐killing capacity, particularly in the early phases of treatment (before day 6), which may be attributed to the inability to continuously supplement T cells in nude mice (Figure 6I‒K). Correspondingly, flow cytometry analysis revealed that STM2457 treatment could reduce CD8^+^ T‐cell exhaustion and enhanced CD8^+^ T‐cell cytotoxicity (Figure S5E,F). In summary, these findings highlight the potential of METTL3 inhibition as a strategy to directly enhance T‐cell function, thereby providing a promising approach for enhancing the efficacy of immunotherapies.

## 4 DISCUSSION

Although ICB therapy has shown clinical efficacy in reactivating the immune system against cancer, only a subset of patients with specific tumour types respond to it.^44^, ^45^ This indicates an urgent need for more effective combination therapy to overcome immune resistance. Studies have shown that knocking out METTL3 or applying METTL3 inhibitors in tumour cells can improve the efficacy of ICB therapy. Specially, targeting METTL3 in tumour cells can increase the interferon response, enhance MHC‐I exposure, and upregulate PD‐L1 both in vitro and in vivo.^14^, ^15^ However, the role of METTL3 in modulating the tumour microenvironment remains poorly understood. In this study, we functionally validated the relationship between T cells targeting METTL3 and anti‐tumour immunity. Inhibition of METTL3 not only promotes the immune response and enhances the immunogenicity of tumour cells, but also reverses T‐cell exhaustion in tumour microenvironment, thereby sensitising ICB‐based immunotherapy.

The major mechanism by which m^6^A affects mRNA is by the recruitment of m^6^A binding proteins, specifically the YTH domain family (YTHDF1‐3 and YTHDC1‐2).^34^ Therefore, identifying the downstream executing YTHs reader protein is crucial to understanding how METTL3 regulates immunotherapy. Among these reader proteins, YTHDC1 depletion leads to early embryonic lethality in mice, similarly to METTL3 deficiency,^46^, ^47^ while YTHDF1‐3 deletion does not.^48^, ^49^ In the cytosol, YTHDF1 facilitates the translational efficiency of target mRNAs,^50^ YTHDF2 accelerates mRNA decay^51^, ^52^ and YTHDF3 promote mRNA translation and degradation.^53^, ^54^ Therefore, the different outcomes of METTL3 disruption may be attributed to the execution of different m^6^A readers. Our data show that METTL3 KO inhibits B16 cells proliferation, while METTL3 inhibition or YTHDF1‐3 KO does not (Figures S1A and S3B). Speculatively, METTL3 KO and inhibition exhibit different effects probably due to dose‐dependent effects. The suppression of tumour proliferation observed in METTL3 KO may be mediated by YTHDC1, while the effect of METTL3 inhibition is mainly mediated by the YTHDF family. From the results of CRISPR‐Cas9‐mediated YTHs protein KO B16 mouse tumour model, we discovered YTHDF2 deletion had a notable inhibition of tumour growth through T‐cell immune system. This effect is analogous to the impact of STM2457 treatment on ICB therapy. Notably, we analysed the MHC‐I gene upregulated by both targeting METTL3 and YTHDF2, and characterised YTHDF2 as an m^6^A effector reader that plays a role in MHC‐I decay. Taken together, YTHDF2 is likely the downstream executed reader following METTL3 targeting, thus playing a pivotal role in the anti‐tumour response.

Mechanically, the depletion of METTL3 has been shown to enhance the response to anti‐PD‐1 therapy by stabilising the IFNγ‐Stat1‐Irf1 signalling axis in colorectal and melanoma cancer cells, and in non‐small cell lung cancer (NSCLC) cancer cells, METTL3 deletion has been observed to destabilise both c‐Myc and PD‐L1 mRNAs.^14^, ^15^ METTL3 also promoted BHLHE41 expression in an m^6^A‐dependent manner in CRC tumour cells, then promotes MDSC migration via BHLHE41‐CXCL1/CXCR2.^55^ Furthermore, METTL3 inhibition within tumour can increase interferon response and enhance MHC‐I exposure.^56^ In summary, current research on METTL3 in immunotherapy primarily concentrates on the impact of RNA m^6^A modification on immune‐related mRNA metabolism in tumours, as well as on enhancing tumour immunogenicity to affect T‐cell function. However, the impact of RNA m^6^A modification on T cells themselves in the tumour immune microenvironment remains unclear.

METTL3 is known to be essential for embryonic development, stem cell differentiation and immune cell homeostasis.^19^, ^46^ Disruption of METTL3 affects the proliferative and differentiative capacity of CD4^+^ T cells, the function of Tregs, and the maturation of T follicular helper cells (Tfh).^18^, ^57^, ^58^ While it may initially appear that METTL3 is not a viable target for clinical applications due to the lethality associated with complete METTL3 KO, but it is important to note that the consequences of METTL3 deletion are not equivalent to those of METTL3 inhibition (Figures 2, 3). In this study, we discovered that inhibiting METTL3 significantly augmented the effectiveness of ICB therapy in both B16 melanoma and MC38 colon tumour models. Analysis from scRNA‐seq revealed that the CD8^+^ T‐cell population exhibited a reduced expression of exhaustion markers and an upregulation of genes associated with mitotic cell cycle progression and T‐cell activation (Figure 5A‒D). Specifically, we observed reduced expression of exhaustion markers and enhanced cytotoxicity in the CD8^+^ T‐cell population following METTL3 inhibition, both in vivo and in vitro (Figures 2, 6 and S5E,F). Therefore, our primary innovation lies in the novel insight that targeting METTL3 in T cells, rather than tumour cells, is essential for maintaining T‐cell persistence and, therefore, potentiates the efficacy of anti‐PD‐1 therapy.

There are some shortcomings in the current academic exploration. We have used STM2457, a pharmacological inhibitor of METTL3, to explore the therapeutic effect of METTL3 inhibition in tumour therapy. However, using only a pharmacological inhibitor makes it challenging to avoid potential off‐target effects. Although we have performed METTL3 KO using CRISPR‐Cas9 and overexpressed WT/mutant forms of METTL3 in tumour cells (Figure S3C). While these additional approaches help to mitigate off‐target effects, they do not fully explain the phenotypic differences between KO and inhibition. Furthermore, the study only employed a pharmacological inhibitor for the study of T cell. On the positive side, we have found that targeting METTL3 enhances the killing effect on human melanoma cancer in vivo, and in vitro experiments showed that METTL3 inhibition can augment the killing ability of human T cells against tumours (Figures 6G and S2C). Thus, further investigation into the effect METTL3 inhibition in the context of immunotherapy for human cancers is warranted.

**FIGURE 7:**
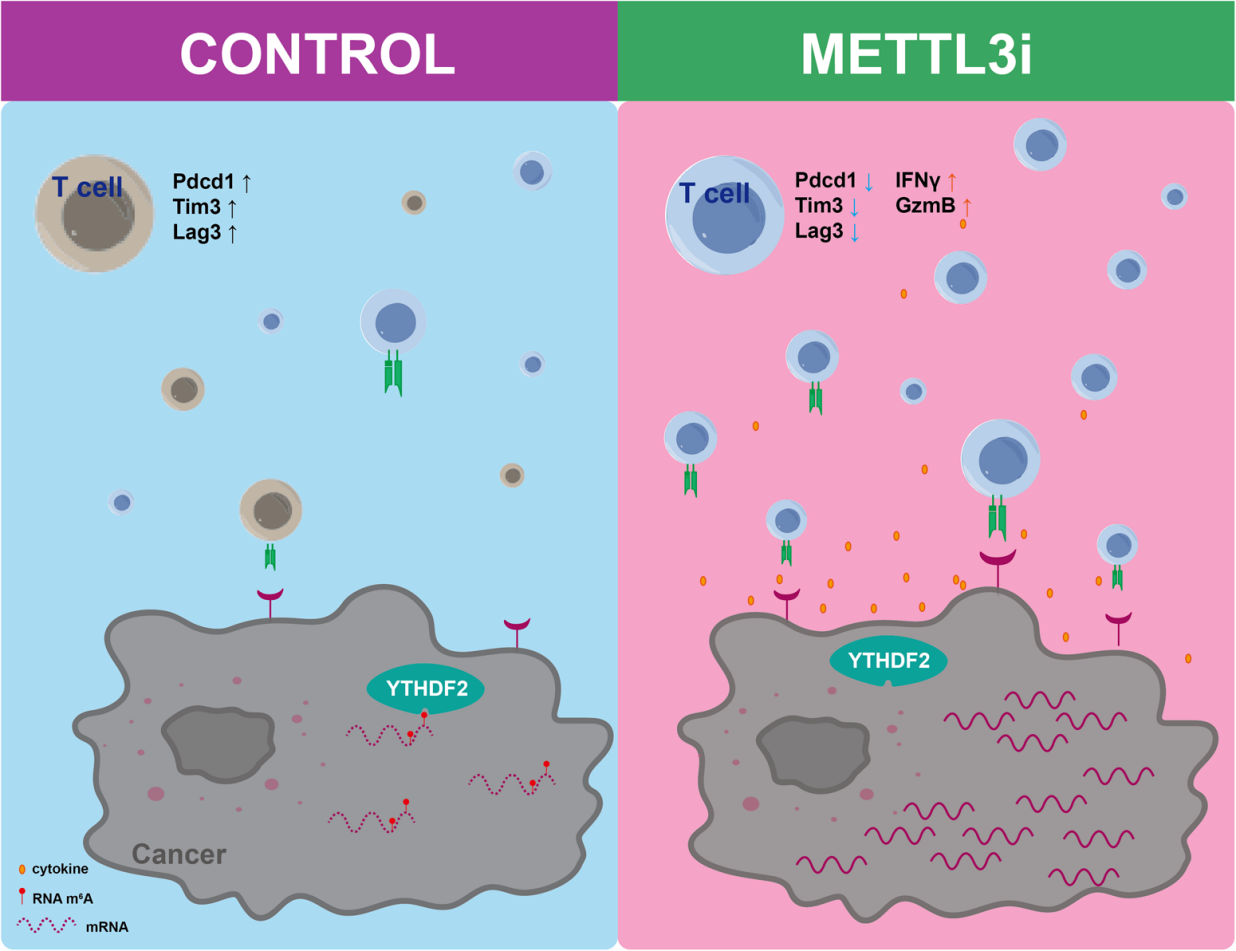
Illustration of the proposed mechanisms of METTL3 inhibition within both tumour cells and T cells in cancer therapy.

In conclusion, our study underscores the therapeutic potential of METTL3 inhibition in combination with anti‐PD‐1 therapy for effective tumour elimination. On one hand, METTL3 inhibition enhances the immunogenicity of tumour cells, promoting their recognition by T cells. On the other hand, it enhances T‐cell persistence by reducing exhaustion and amplifying cytotoxic capabilities (Figure 7). Consequently, targeting METTL3 with an enzymatic inhibitor can achieve a synergistic immunotherapeutic effect, targeting both the tumour cells and enhancing the vigor of T‐cell responses.

## AUTHOR CONTRIBUTIONS

K.W. and S.L. performed the major experiments. J.H., and G.H. performed the bioinformatics analysis. K.W. and L.J. performed the RNA‐seq experiments. H.L. performed the scRNA‐seq experiments. K.W., and S.L. performed cell culture experiments. K.W., S.L., H.D. and T.T. accomplished in vivo studies. L.J., S.L., M.H., G.L., S.C., H.W., and J.J. contributed to the work. K.W, J.H. and S.L. wrote the manuscript. HY.W., HL.W., X.K. and J.C. helped to improve it. HL.W., J.H. and K.X. conceived and supervised the entire study.

## CONFLICT OF INTEREST STATEMENT

The authors declare they have no conflicts of interest.

## Supporting information



Supporting Information

Supporting Information

Supporting Information

## Data Availability

The data that support the findings of this study are available from the corresponding author upon reasonable request. The datasets generated and analysed during the current study are available in the BioProject accession number PRJCA023039.
