# Supplementary material for: Targeting METTL3 as a checkpoint to enhance T cells for tumour immunotherapy
Source: Clin Transl Med. 2024 Nov 20;14(11):e70089. doi: 10.1002/ctm2.70089 (PMC11578931; doi:10.1002/ctm2.70089)
Supplement: Supplementary file 2 — Supporting Information [file CTM2-14-e70089-s002.docx]

**
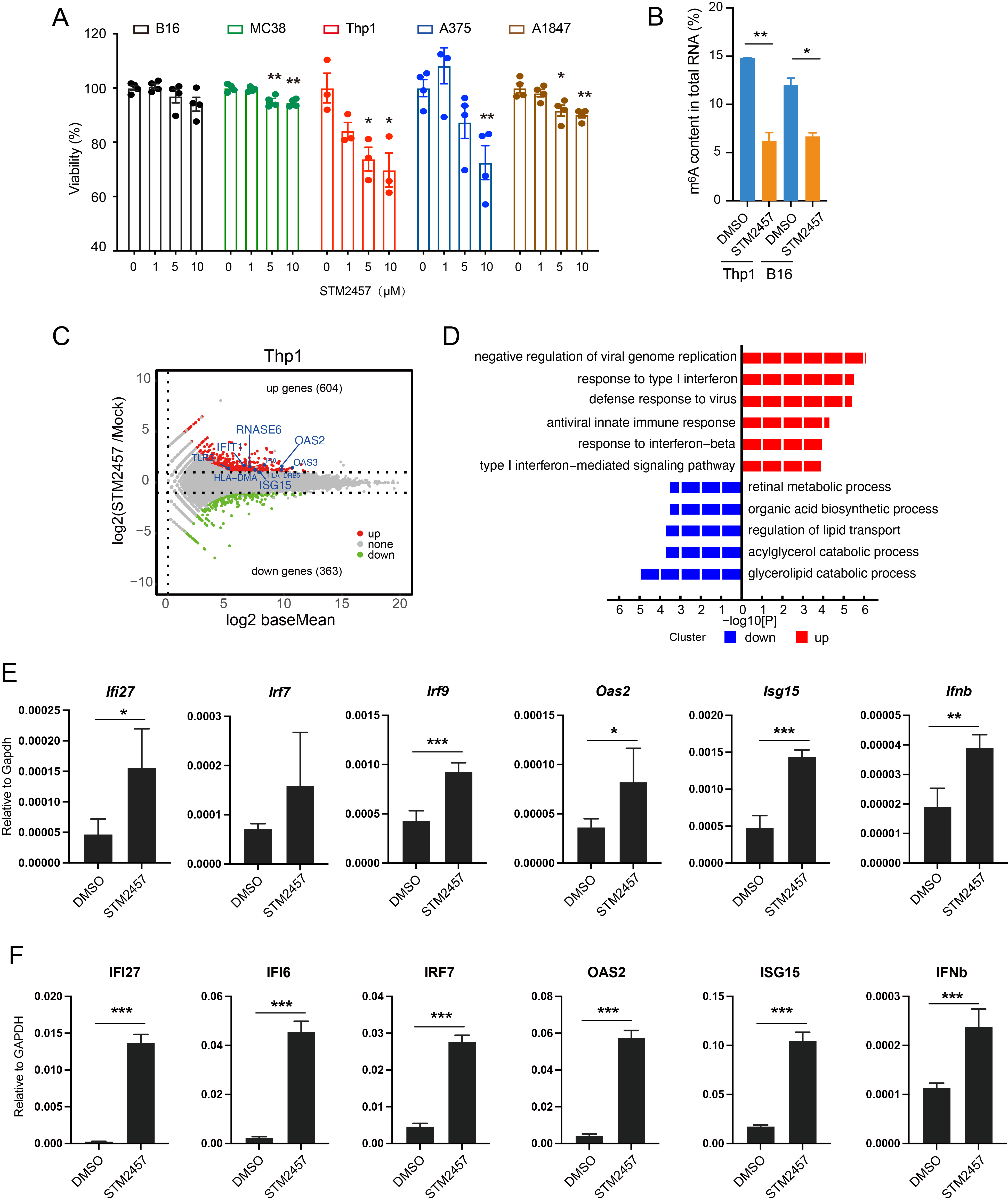
**

**Figure S1. Related to Figure 1.**

(A) Cell viability assay showing the viability rates of B16, MC38, THP-1, A375, and A1847 cells exposed to various concentrations of STM2457(n=3 or 4, biological replicates; error bar, SD; *p<0.05, **p<0.01).

(B) The m6A content in THP-1 or B16 cells was determined using LC-MS/MS. (n=2, biological replicates; error bar, SD; *p<0.05, **p<0.01).

(C) Volcano plot showing differentially expressed genes (DEGs) from RNA-seq analysis of THP-1 cells treated with STM2457 versus Mock treatment. Red dots represent upregulated genes and green dots represent downregulated genes.

(D) GO analysis of DEGs in THP-1 cells treated with STM2457 versus DMSO.

(E and F) Real-time quantitative PCR (RT-qPCR) analysis of transcripts of selected immune genes in B16(E) and THP-1(F) cells treated with STM2457 versus DMSO. (n=2, biological replicates; error bar, SD; *p < 0.05, **p<0.01, ***p<0.001).

**
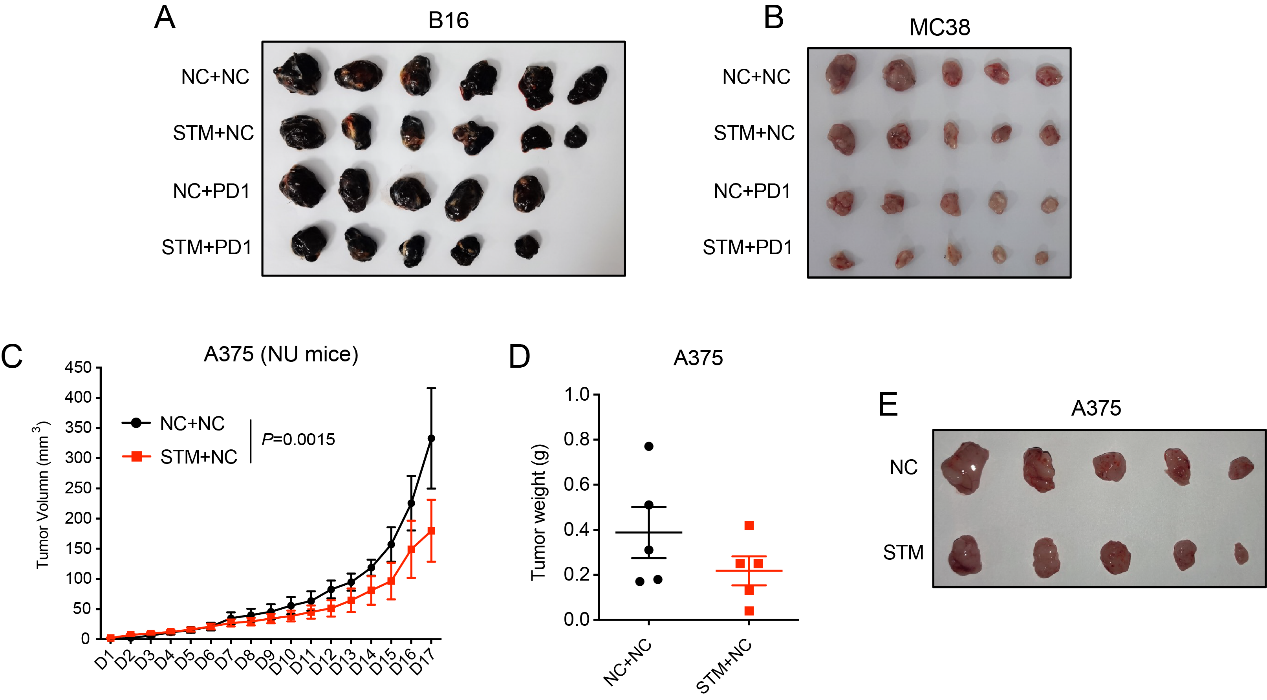
**

**Figure S2. Related to Figure 2.**

(A) Tumor image of B16 tumor engraftment C57BL/6 mice treatment by PD-1 Ab in combination with STM2457.

(B) Tumor image of MC38 tumor engraftment C57BL/6 mice treatment by PD-1 Ab in combination with STM2457.

(C) Tumor growth curves of A375 tumor engraftment NU mice treatment with STM2457 versus NC (n = 5; error bar, SEM; Two-way ANOVA).

(D) Tumor weight of NU mice after treated with STM2457 versus NC. (n=5 of 2 biological replicates, SEM; unpaired t-test)

(E) Tumor image of A375 tumor engraftment NU mice treatment with STM2457 versus NC.

**
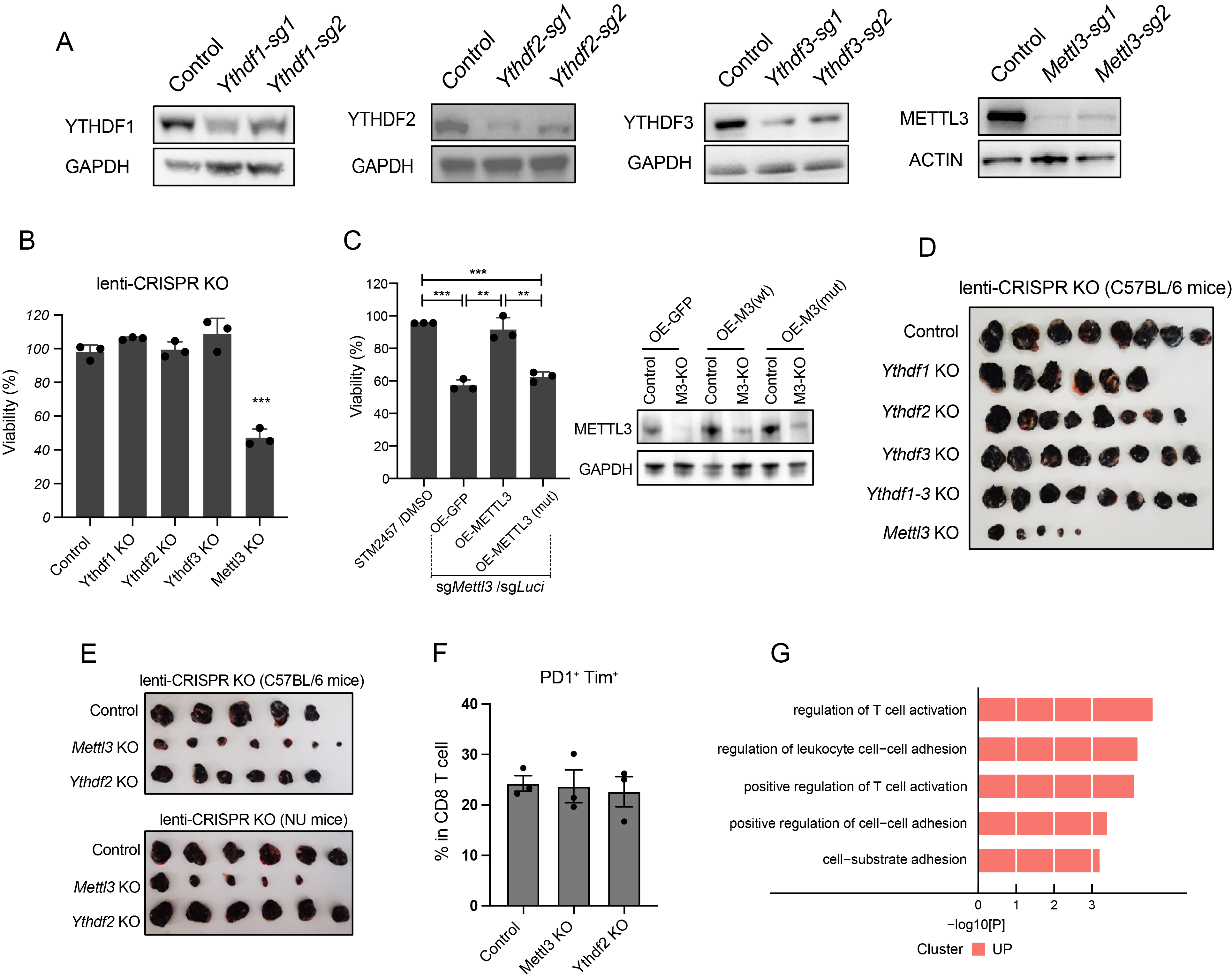
**

**Figure S3. Related to Figure 3.**

(A) Western blot analysis of the expression of YTHDF1-3, METTL3 proteins in B16 cells genetically modified by target sgRNAs.

(B) Cell viability assay showing percentage of B16 cells survival. B16 cells were genetically modified by CRISPR/Cas9 to knock out Ythdf1-3, Mettl3, or luciferase (as a control) genes (n=3, SEM; unpaired t-test, ***p<0.001).

(C) Cell viability assay showing the viability of STM2457 treated B16 cells and overexpressed wild-type (WT) or catalytically dead mutants (DPPW 395-398 APPA) of *Mettl3* in METTL3-KO B16 cells (n=3, SEM; unpaired t-test, *p < 0.05, **p<0.01, ***p<0.001), corresponding Western blot analysis is on the right panel.

(D) Tumor image of B16 tumor engraftment C57 mice. B16 cells were genetically modified by CRISPR/Cas9 to knock out Ythdf1-3, Mettl3, or luciferase (as a control) genes. n = 8 tumors per group.

(E) Tumor image of B16 tumor engraftment C57BL/6 and Nude mice. B16 cells were genetically modified by CRISPR/Cas9 to knock out Ythdf2, Mettl3, or luciferase (as a control) genes. n = 7 tumors per group.

(F) Flow cytometry showing the ratio of PD1+Tim+ cells in CD8+ T cells in B16 tumors. Comparison shown between Control, Mettl3 KO, and Ythdf2 KO conditions.

(G) GO analysis of DEGs in B16 cells after Ythdf2 knockout.

**
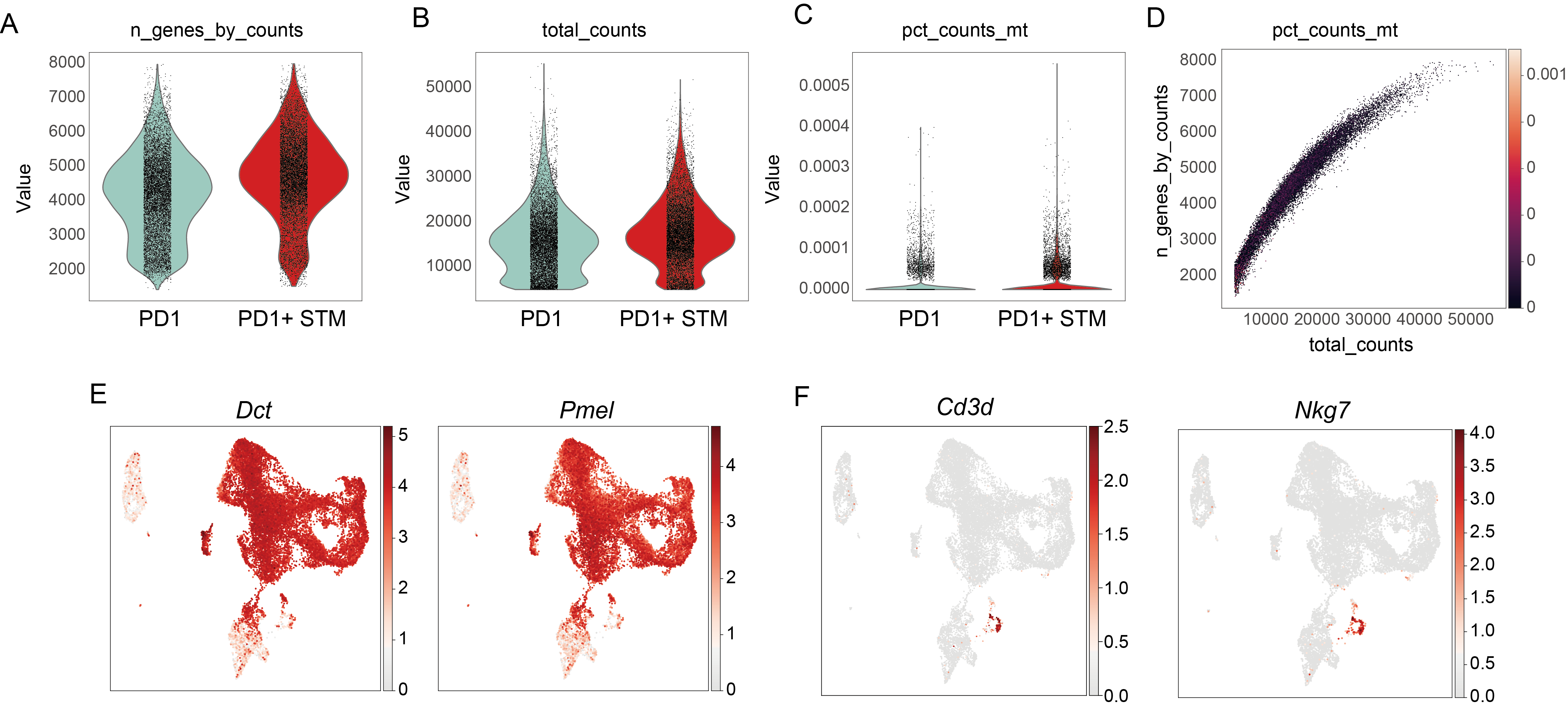
**

**Figure S4. Related to Figure 4.**

(A-C) Violin plot showing the number of genes expressed (A), total counts (B) and the percentage of counts in mitochondrial genes (C) per cell.

(D) Scatter plot depicting the correlation between the number of genes expressed and total counts detected in the same cell, color represented the percentage of counts in mitochondrial genes.

(E and F) UMAP plots showing the expression of maker genes for particularly cell types. Gene expression levels are indicated by shades of red.


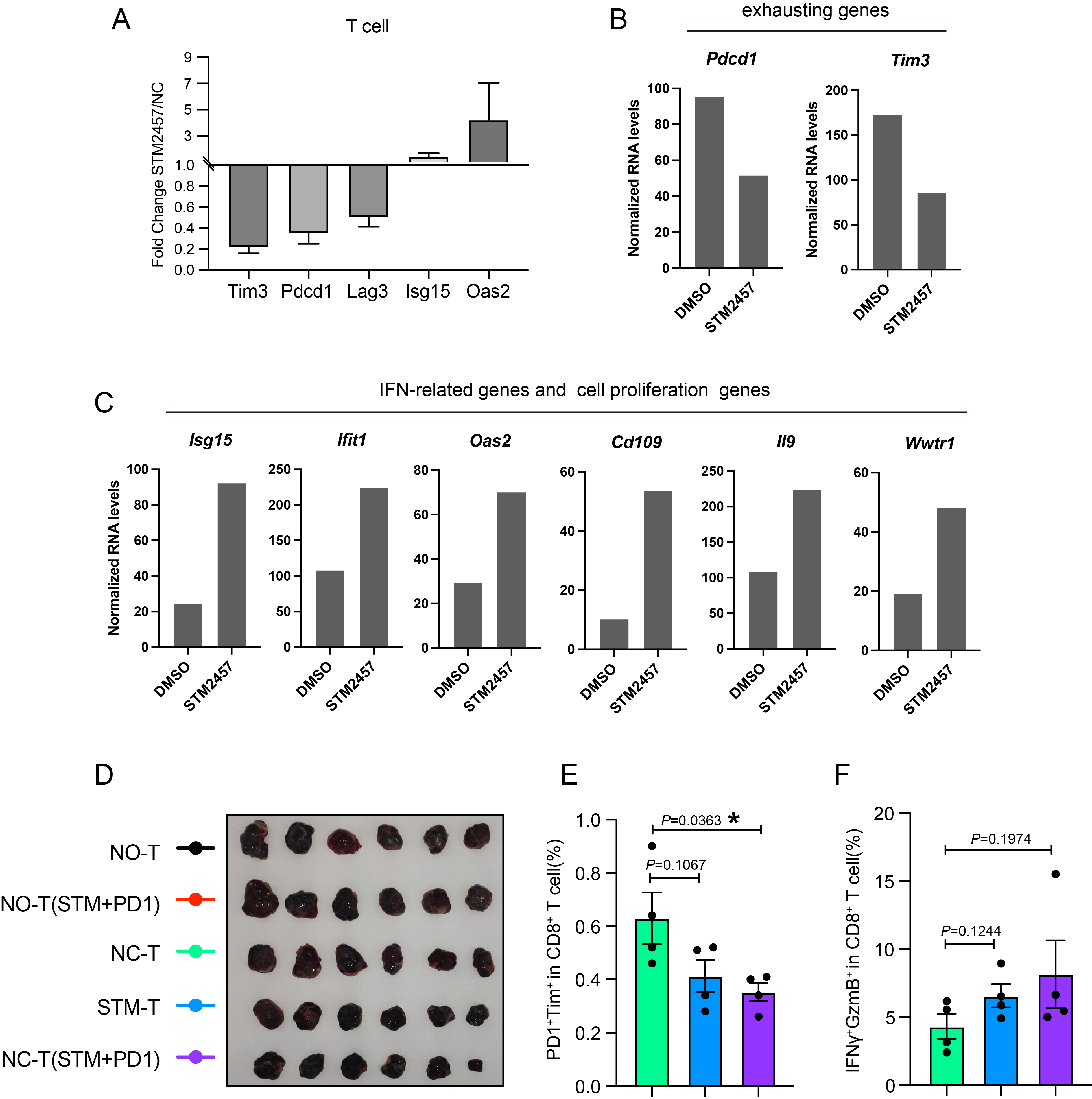


**Figure S5. Related to Figure 6.**

(A) Fold change of transcripts of exhaustion and representative markers in T cell treated with STM2457 vs. Mock (n=4, biological replicates; error bar, SEM).

(B and C) RNA-seq analysis of the expression levels of exhausting genes (B) and IFN related and cell proliferation genes (C) in T cells after STM2457 treatment.

(D) Tumor image of B16 tumor engraftment Nude mice as described in Fig. 6H.

(E and F) Flow cytometry showing the ratio of PD1+Tim3+ cells in CD8+ T cells in B16 tumors(E); the ratio of IFNγ and GZMB positive cells in CD8+ T cells in B16 tumors(F). Tumors were treated as described in Fig. 6H. (n = 4, error bar, SEM; unpaired t-test, *p < 0.05)
